# Mourning the lost: A social actor analysis of gender representation in the @FacesofCovid’s tweets

**DOI:** 10.3389/fpsyg.2022.1042621

**Published:** 2022-12-21

**Authors:** Shrouq Almaghlouth

**Affiliations:** Department of English, College of Arts, King Faisal University, Al Hofuf, Saudi Arabia

**Keywords:** mourning discourse, Twitter, COVID-19, corpus linguistics, social actor representation

## Abstract

When COVID-19 swept the world at the end of 2019, it changed life as we knew it. With about 600 million positive cases (both recovered and active) and approximately 6.5 million deaths due to the disease, people worldwide have been affected physically, psychologically, economically, and socially by the pandemic. Amid such difficult times, @FacesofCovid—a Twitter account with more than 150,000 followers—was launched in March 2020 with the mission of honoring the lives of those lost to COVID-19 instead of presenting them as mere statistics. The account is a demonstrative example of the mourning genre as primarily exhibited through concise tweets grieving the deceased. As such, it offers a novel case of a public online mourning platform through microblogging, an understudied research area that merits further examination. A self-built corpus of 280,536 words was built from more than 7,000 tweets on the public account. The analysis presented in this paper focused on how people are constructed in the language of their loved ones as they are mourned through these tweets. Drawing on insight from van Leeuwen’s social actor representation and corpus linguistics, the analysis was conducted using the #LancsBox corpus processing software package. The findings indicated that gender asymmetry persists within this corpus. Therefore, this paper adds to the rich body of literature documenting gender imbalance across different genres and domains. Men are far more present than women and are constructed through functionalization for the most part, whereas women are less functionalized and represented primarily through relational identification. In light of this, it is argued that while sometimes, gender asymmetry can intentionally be ideologically loaded and may serve hidden agendas, at other times, it may inherently and subconsciously be passed on through spontaneous language use.

Each person’s grief is like all other people’s grief; each person’s grief is like some other people’s grief; and each person’s grief is like no other people’s grief.

—William Worden (2009, p. 7).

## Introduction

Nearly 3 years after the novel coronavirus 2019 (COVID-19) first swept the world, humanity is gradually regaining its feet. The pandemic’s rather apocalyptic arrival brought with it a shadow pandemic of dysfunctional grief worldwide (Neimeyer et al., 2021). Not only was this expressed directly through the tremendous loss of life accompanying the pandemic’s arrival but it was also tangibly illustrated by the ever-expanding amount of indirect causalities at the psychological, mental, economical, and social levels. It is estimated that each death caused by COVID-19 affected approximately nine people (Verdery et al., 2020). Undoubtedly, this—coupled with the radical restrictions imposed upon almost every aspect of life as we know it (Lee et al., 2021)—will mark future generations (Gotlib, 2021) as they try to unfold its audacity. Various forms of support are a critical need for bereaved family members. Therefore, such grief should be presented as a pressing public health issue that is no less critical than the pandemic deaths (Morris et al., 2020). In fact, some scholars have argued that COVID-19-related grief is even more severe than other types of grief (Lee et al., 2021). However, grief can be perceived as a paradoxical construct with both universally and individually unique features. Such a view does not come across as simple or straightforward; therefore, this grief should be addressed with a multidisciplinary perspective and *via* different sources.

A significant form of support has been made readily available through the affordances of the internet *via* the creation of virtual support communities with different motivations. One such example is @FacesofCovid (FoC), a Twitter account with the mission of honoring the lives of those lost to COVID-19 in the form of micro-eulogies. This study uses a combination of corpus tools and social actor representation analysis to examine how these tweets construct the deceased concerning gender-based categorization. This section offers an up-to-date background on relevant research in discourse studies. Given the interdisciplinary core of the current paper, relevant areas within the literature of discourse on grief and gender as socially constructed constructs are reviewed. This information is linked to the hybrid nature of microblogging as a genre as well as the theory laying the groundwork for social actor representation (van Leeuwen, 1996, 2008) as an analytical framework.

Grief is not merely an internal process; it is often presented in the relevant literature as an intricate, socially constructed construct (Neimeyer et al., 2014). This suggests that grief can be presented from a constructionist perspective, which denotes that—despite its universal and biological roots—grief can also be subjected to socially and culturally shared norms and values. Mourning is a specific kind of grief that follows the death of a loved one. Neimeyer et al. (2014, p. 487) linked mourning, as a communicative event, to narrative activities since the mourner becomes involved in “storying” processes in search of the meaning behind their loss. However, this is not the sole function achieved by such an act, as indicated by the rich body of related research. Therefore, while people mourn to make sense of fate (Davis et al., 2016) or process their loss (Böhme, 2017), they also resort to it to ease their grief (Harris, 1999), foster familial resilience (Barboza et al., 2022), create bonds with other bereaved people (Church, 2013), build communities (Jensen et al., 2003), offer emphatic support (Cupit et al., 2021), give advice (Foss, 1983), or even call for action (Jensen et al., 2003). However, it is clear that the primary function of mourning is honoring and praising the deceased (Kent, 1991), a process that sometimes idealizes them (Hayes, 2016). This multifunctional conception transforms grief, and mourning in particular, into an event of its own (Merrin, 1999), thus granting grief discourse key significance in social constructionist research.

While mourning might take several communicative forms, eulogies are the most prominent. Eulogies are “a genre, or specific type, of the rhetorical form” (Davis et al., 2016, p. 317) serving a multiplicity of purposes—as examined above—that are inevitably dictated by varying factors across time and space. In the conventional sense, eulogies are often delivered by relatives, friends, or clergy members (Stevens, 2008), thus making them an instance of ceremonial discourse. While a plethora of research on grief discourse is predominantly based on psychological theory (e.g., Hayes, 2016; Lessard et al., 2016), eulogies have not been sufficiently addressed in the recent linguistic literature. For instance, Mohammed and Khudher (2018) contrasted the long history of eulogies, which can be traced back to the ancient Greeks, with the limited number of recent research works in the genre. Nevertheless, several interdisciplinary discourse studies can be identified, primarily those examining the eulogies of celebrities and politicians (e.g., Sanderson and Hope Cheong, 2010; Szeligowska, 2014; Williams, 2014). However, discourse works with corpus-based motivations are almost non-existent, with the exception of Slavickova’s (2013) work on presidential Memorial Day speeches in the US. This highlights the need for motivating discourse studies that adopt a corpus-based approach as they analyze eulogies addressed in memory of non-famous individuals.

Regarding gender-related research, often, gender asymmetry and sexism predominate. As such, more awareness toward such asymmetry is needed given that gender is culturally constructed and exhibits variations across different contexts (Baker, 2003), unlike sex, which is determined biologically. This constructionist take on gender, however, has not always been approached as such in the relevant literature, and there has been a dispute over the nature of gender. Thus, it is also possible to consider gender from an opposing perspective, i.e., the essentialist take on gender, which presupposes a predominantly genetic mapping of how it can be perceived. Baron-Cohen (2004), for instance, highlighted the biological differences between female and male brains, stating that the former is hard-wired predominantly for empathy while the latter is more biologically geared toward understanding and building systems. Similarly, Bem’s (1974) gender theory and sex role inventory, which has gained popularity over the last few decades, presupposes different social and psychological constructions of men and women based on biological classification. The problem with such essentialism, however, is that it overlooks the potential of context, both social and psychological, in shaping the roles expected to be played by both genders and, consequently, how this is mirrored in discourse.

A more inclusive perception of gender is highlighted in Eagly and Wood’s (2012) social role theory. Within this conception, biology works alongside psychology to determine the gendered performance of social roles. This signifies the constructionist take on gender while still adhering to biological determinism (Wood and Eagly, 2012). Several other theories, however, reject the notion of the gender binary altogether (Hyde et al., 2019) and argue for problematizing biologically based gender construction. A well-known example is Butler’s (1999); (Butler, 2006) call for the notion of gender trouble within the last century, which has shaped much of the research in that direction (Morgenroth and Ryan, 2018). The current paper adheres to the more inclusive view of gender established above. By highlighting the biological mapping of gender while still acknowledging the impact of social shaping in its construction, the social constructionist aspect of the discourse is also pinpointed and maintained throughout the analysis.

In gender-based discourse studies, corpus tools have been extensively applied since the 1980s. With the emergence of big data and natural language processing, works such as Kjellmer’s (1986) started to appear and document gender asymmetry in language. In Kjellmer’s study, the Brown corpus revealed more instances of *he* than *she*. By the same token, to name a few, Cameron (1992), Sutton (1995), Romaine (1999), Holmes and Sigley (2001), Pearce (2008), Baker (2010), Caldas-Coulthard and Moon (2010) and Moon (2014) have conducted other corpus-based studies in which similar asymmetries were documented between men and women. As more corpus research is conducted, such imbalances continue to appear, documenting the fruitful application of corpus tools in revealing even subtle gender differences in language (Carroll and Kowitz, 1994). Indeed, irrespective of the advancements made in advocating women’s rights and women’s empowerment, stereotypical portrayals and underrepresentation of women persist (Vu et al., 2018). A detailed examination of the relevant literature primarily documents that women are less present in language use (Baitinger, 2015). The rich body of research in this particular area reveals this to be the case across different areas of research in discourse studies, including political discourse (Grunenfelder, 2013), academic writing discourse (Brooke, 2020), literary discourse (Eberhardt, 2017), and even women’s empowerment discourse (Brun-Mercer, 2021). However, within the discourse on grief and mourning, and in eulogies, in particular, there seems to be a gap in gender-based analysis. Therefore, the current study will contribute significantly to this body of research.

Note that as a corpus-assisted discourse study, interdisciplinary analysis and multiplicity of frameworks appear to be the norm. With this in mind, the current study has another cornerstone, which follows the constructionist nature of gender and grief as social constructs. In particular, the social actor representation analytical framework (van Leeuwen, 1996, 2008) seems fit for purpose given the interplay of such factors. According to the latter (p. 67), this model presents a socio-semantic perspective on discourse, bringing together “what linguists tend to keep separate.” In such a conception, meaning is deeply rooted in culture rather than language or individuals. As it stands, the model offers an extensive list of transformations to account for how diverse social actors are constructed in text in a hierarchical fashion. The framework starts with inclusion and exclusion across varying levels of representation and through a diversity of role allocation. As a result of its breadth, the application of social actor representation in the literature on discourse studies seems relatively common, including works on execution (Utama et al., 2020; Chaemsaithong, 2021), terrorism (Rasoulikolamaki and Kaur, 2021), disability (Ang and Yeo, 2018), and politics (Asad et al., 2019; Surjowati, 2020; Diamante, 2021), as well as those with corpus-based motivations (García-Marrugo, 2013; Bakar, 2014; Fadanelli et al., 2020).

The above discussion offered a concise background on the different cornerstones that ground the current research work. This was achieved by focusing on discourse on grief, mourning, and eulogies while highlighting relevant literature on gender construction through discourse as well as social actor representation as a socio-semantic framework to underpin discursive constructions. However, the remainder of this section tailors the discussion to address the virtual component of this study. To illustrate, the plethora of research on online discourses asserts the immediacy and easy availability of online practice in a time- and effort-consuming manner (van Leeuwen et al., 2022), as social media posting is quite common nowadays (Cupit et al., 2021).

Scholarly interest in microblogging, a form of social media posting, has skyrocketed since its conception (Liu et al., 2011). Zappavigna (2014, p. 209) defined microblogging as “the act of posting short character-constrained messages to the Internet.” The act comprises unique features, such as a constrained message length, support for mobile phones, and access to open and free online publishing (Oulasvirta et al., 2010); it is a distinct form of two-way communication (Morehouse and Crandall, 2014). Despite being a novel genre in the new millennium, microblogging can be linked to older forms of human communication due to its inherently interpersonal nature. Zappavigna (2014), for instance, linked it to the Firthian perspective (Firth, 1964, p.112), contending that the “promotion, establishment and maintenance of communion of feeling is perhaps four fifths of all talk.” This also corresponds to Malinowski’s (1972) notion of phatic communion. With this in mind, the utilization of microblogging as an outlet to communicate feelings seems inevitable. However, this may not be the only gratification associated with microblogging.

This notion can be considered in light of the uses and gratification theory, a well-known theory of communication in media psychology literature. Emerging in the 1940s and expanding in the 1970s, the uses and gratifications theory suggests that people prefer to use certain media platforms, as they expect such use to satisfy certain gratifications consistent with the use of these platforms (Ruggiero, 2000). While this theory places its primary attention on the consumer of such platforms more than the conveyed messages, certain implications can be drawn here. In particular, while most relevant literature on discourse studies acknowledges the interpersonal functions expressed in microblogging, research in other disciplines denotes other gratifications. For instance, the use of microblogging within educational research has proven to offer language learners increased engagement and expanded knowledge (Gant and Hadley, 2014). In another contrastive study (Li et al., 2019), the use of different cultural contexts and microblogging applications, such as Twitter and its Chinese counterpart, Weibo, led to different gratifications for the followers of sport organizations. In particular, while Twitter microbloggers were microblogging to express their support of their teams, Weibo followers were microblogging to obtain more information or pass the time.

By the same token, another contrastive study reported different gratifications satisfied by the users of Twitter and Weibo within the microblogging discourse of disaster following two major industrial accidents in the United States and China (Wu and Montgomery, 2021). Here, Twitter users were utilizing microblogging to create a supportive community for those suffering after the incident much more than Weibo users. In this sense, it is possible to see the link between research on microblogging discourse and what Palen and Hughes (2018) referred to as crisis informatics. In crisis informatics, the analyst is concerned with the communicative response of people on social media following a given crisis or disaster. Within grief discourse, in particular, microblogging is not used solely for sense-making, bonding, or creating communities but is also used for other, unexpected functions. This is evident, for instance, when examining the analyses of grief discourse regarding deceased celebrities. In their examination of more than 50,000 tweets following the death of Michael Jackson, Lee and Goh (2013) revealed categorizations that were not normally associated with grief discourse, such as spamming and the spreading of rumors or hatred.

This diversity of perspective should not undermine the dominant interpersonal vein established earlier within the discourse on microblogging. Various studies have shown that many people are increasingly inclined to share their feelings, whether positive or negative, online (Brubaker et al., 2012). With this in mind, Selfridge and Mitchell (2021) argued that the private becomes public on social media platforms, while Morehouse and Crandall (2014) highlighted the move from private to public expression online. To elaborate, with the advent of microblogging, the expression of grief, which used to be an almost exclusively private process, is gradually being transformed into a public process. To understand this perspective, the affordances of social media platforms that support the creation of virtual communities around a particular topic or event should be considered. Zappavigna (2014, p. 211) linked this to the notion of “ambient” affiliation, in which a direct interaction, such as a conversation, between the participants in that community is not necessarily presumed. Such affiliation can also be achieved by simply discussing a particular topic around the same time, which was the case with FoC. As people sent their mourning tweets to the account to be posted or retweeted, they were allowing even those who were unrelated to the deceased to express their reactions since they were ambiently affiliated. Morehouse and Crandall (2014) drew attention to the fact that what used to be considered trespassing on people’s private lives is now being normalized. Despite cyber security and privacy risks (Oukemeni et al., 2019), participants and followers of such platforms continue to publicize their private content. This move away from privacy can also be linked to another inherently available social media tool, i.e., the use of hashtags. With hashtags, microbloggers intentionally link their content to an even wider audience, creating what Boyd (2011) referred to as a networked public in the sense that even the “ordinary” becomes visible to everyone (Oulasvirta et al., 2010, p. 238).

Considering the above discussion of grief as an internal, social, and cultural construct, it could be proposed that the inherently intricate nature of grief denotes that the boundaries between what is private and what is public are blurred; the connection between these two should not be conceptualized as a binary but rather as a continuum. Thus, it is not surprising that online grief is common during times of stress or disaster. In fact, Kakar and Oberoi (2016) formalized it as a death ritual in our modern world. However, this move should not be seen as a rejection of conventional communicative forms of grieving, such as traditional face-to-face eulogy delivery (Lingel, 2013). Instead, it is a complementary parallel form of grieving in response to modern-day affordances and the availability of social media in discourse construction and circulation. This idea of complementary parallelism is supported by the fact that while traditional eulogies offered during funerals can help provide closure, online mourning through social media platforms can be shared with a wider audience and is definitively engraved virtually as endless (Morehouse and Crandall, 2014).

This has led to the emergence of “digital memorialization” as a form of online grief (Church, 2013, p. 184). The latter, drawing on Roberts’s (2004) virtual cemeteries, explores the links between the material and demolishing event of death and the ever-existing display of online grief through the affordances of social media. Such digital memorialization has been the subject of attention in several discourse studies utilizing a variety of semiotic frameworks, such as the works by Church (2013), Visa and Briones-Vozmediano (2020), and Cupit et al. (2021). However, the current paper proposes that the utilization of microblogging through tweets on Twitter in the form of concise eulogies results in the hybrid genre of micro-eulogy. In micro-eulogies, the person delivering the eulogy—in this case, the original writer of the tweet—produces a short text that corresponds to moves identified in the traditional eulogy genre while being confined by the character count limitation imposed by Twitter. This inevitably produces a more concise and content-based text while devaluing rhetorical and stylistic features often associated with the eulogy genre.

This detailed review is meant to pave the way toward an understanding of the rationale behind the current study’s data selection, collection, and analysis methodology. Considering the problem stated at the outset of this paper, the following research questions (RQs) frame this study:

RQ1: Using corpus tools, which linguistic constructions are established most frequently in the FoC? Which nouns and adjectives are predominantly used from such perspectives?

RQ2: Using corpus tools, what collocational patterns are associated most frequently with gendered terms in the FoC?

RQ3: What kinds of social actor representations are associated most frequently with gendered terms in the FoC?

## Methodology

The affiliation between corpus linguistics and discourse studies has strengthened since the emergence of automatic language processing within the last century, and the bond between these fields continues to grow as more published research advocates such an affiliation (Liu and Afzaal, 2021; Afzaal et al., 2022; Du et al., 2022). Indeed, the utilization of corpus tools along with other linguistic frameworks for discourse studies has helped overcome certain subjectivity issues that are often brought up when only alternative approaches, such as content analysis, are implemented (Baker and Levon, 2015). This is not to say that empirical data in both quantitative and qualitative forms cannot be obtained through such approaches; however, automatizing them through corpus tools significantly improves objectivity (Afzaal et al., 2019). In light of this strong bond and given the RQs, a corpus of 280,536 words was created from the official FoC Twitter account. More than 7,000 tweets posted between March 2020 and February 2022 were extracted from Twitter Premium API using TrackMyHashtag, a third-party tool. The data exhibited several informal features associated with microblogging genres, such as the use of symbols (Herdagdelen, 2013), warranting their cleaning. The data were then transformed into corpus-friendly plain texts. Later, the texts were imported into the #LancsBox (Brezina et al., 2021) corpus processing software. These tweets were already publicly and freely available online, and the account creator, @alexjgoldstein, was informed about this project and approved the analysis of the tweets as a public source. In accordance with the copyright concerns, no tweets have been reproduced here since the processes undertaken in corpus-based works often transform such texts into disjointed words and phrases in search of linguistic patterns. In addition, no names or identities have been disclosed or reproduced.

**FIGURE 1:**
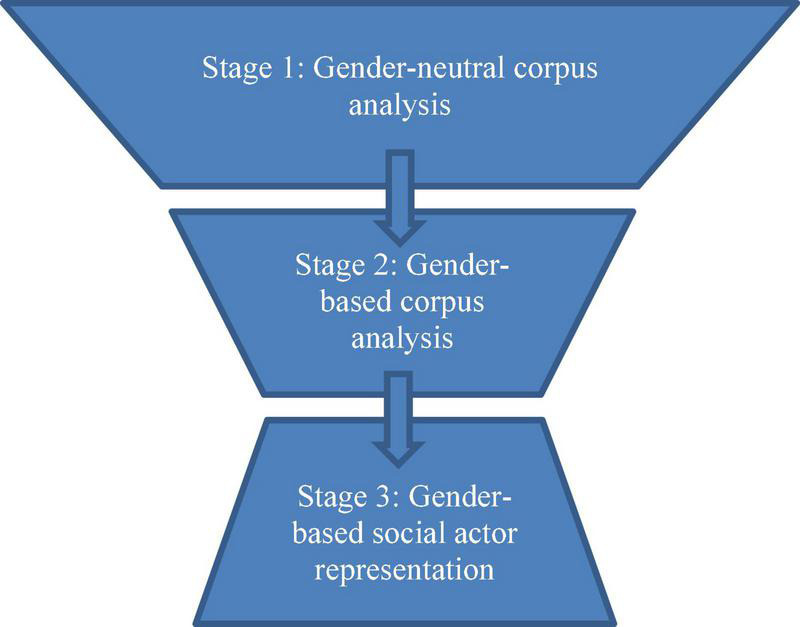
Flowchart of the procedure.

**FIGURE 2:**
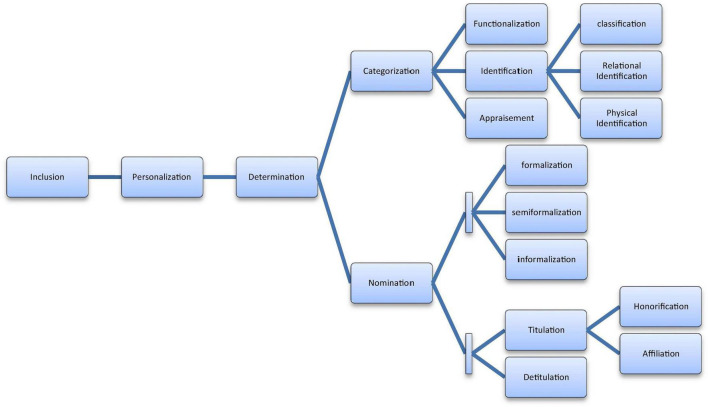
Social actor representation, adopted and modified from van Leeuwen’s (2008, p. 52).

The study was designed to comprise three stages, each of which corresponded to one of the RQs, allowing for triangulation (Baker and Levon, 2015) of the analysis (Figure 1). To elaborate, stage 1 was devised to address RQ1, stage 2 to address RQ2, and stage 3 to address RQ3. In stage 1, the analysis utilized a gender-neutral corpus analysis in a top-down fashion. By examining the corpus as a whole using the *Words* tool in #LancsBox and identifying the most frequent nouns and adjectives, this stage aimed to reveal descriptive information concerning the general themes occurring in the FoC tweets. Establishing the general linguistic representations in the FoC tweets, irrespective of gender, was necessary to proceed with the following stages. First, this was to highlight linguistic constructions that were rather common to both genders and associated with eulogies in general; second, this allowed for further investigation of the interpersonal aspect of microblogging. In stage 2, the same top-down approach was applied, as the *KWIC* and *GraphColl* tools were used to examine both the concordance lines of the gendered terms in the FoC tweets, such as *he, she, man*, and *woman*, and their collocations. In stage 3, manual analysis was adopted. Using the RANDOM function in Excel, 100 random tweets mourning men and 100 random tweets mourning women were examined in detail. This process was conducted in a bottom-up fashion within the framework of social actor representation (van Leeuwen, 1996, 2008). As stated previously, this is an extensive framework; therefore, only certain representations bearing significance to the current research problem were examined. Categorization and nomination were chosen as primarily substitution levels (van Leeuwen, 2008, p. 40-53). Categorization can be expressed through functionalization (e.g., *guardian* or *pianist*), appraisement (e.g., *good, bad*, or *admired*), or identification. Identification can be further expressed through classification (e.g., *Muslim* or *woman*), relational identification (e.g., *friend* or *mother*), or physical identification (e.g., *blonde* or *chubby-cheeked*). Nomination can be expressed through formalization (surname only), semi-formalization (given name and surname), or informalization (given name only). It can also be expressed through titulation—through honorification (e.g., *Dr*.) or affiliation (e.g., *Auntie*)—or detitulation (i.e., when no titles are used). Figure 2 below is adopted and modified from van Leeuwen’s (2008, p. 52).

## Results

### Stage 1

The analysis started with taking a gender-neutral perspective on the dataset and top-down processing of the FoC tweets. Using #LancsBox, the dataset was processed *via* the *Words* tool to generate the most frequently used words in the tweets. However, this process resulted in an extremely large amount of types (26,167 words) that could not be analyzed in total for reasons concerning practicality, significance, and indication. Consequently, the option for list sorting on #LancsBox was shifted from types to lemmas. A lemma in corpus linguistics refers to a word’s head entry in a dictionary (Baker, 2006); for instance, the words *play, plays, played*, and *playing* belong to the same lemma (*play*). By sorting the list in such a way, variations in the same words were combined to reduce duplication. Then, the *Words* tool tagged these lemmas for their parts of speech as they were sorted. Accordingly, the top 50 nouns and top 50 adjectives in the most frequent lemmas are identified in Table 1. Verbs were excluded from this section, as they bore less relevance to the RQs. Note that like all corpus-assisted discourse studies, function words, e.g., articles, prepositions, and pronouns, appeared more frequently in this list than content words, e.g., nouns, adjectives, and verbs. Function words usually dominate the top places in frequency lists; as a result, the first content word in the FoC list (i.e., *die* as a lemma) came in fifth place after *of, a, the*, and the conjunction *and*. However, function words were excluded at this stage for the sake of analysis and clarity of presentation.

**TABLE 1:** The 50 top nouns and 50 top adjectives in the FoC.

Nouns				Adjectives			
Lemma	Frequency	Lemma	Frequency	Lemma	Frequency	Lemma	Frequency
Covid/Covid19	5,593	Florida	346	More	588	Wonderful	99
Rt	2,537	Community	341	Beloved	517	Long	97
@FacesofCovid	1,645	Dad	335	Many	463	Generous	96
Family	1,310	Dec.	330	Good	432	Positive	94
Year	1,024	May	325	Great	280	Former	89
Life	961	Love	323	Loving	234	Single	88
April	936	Husband	323	Kind	198	Full	84
New	707	Teacher	315	Much	197	Past	84
One	665	Man	308	Other	176	Funny	82
Day	650	Grandchild	304	Local	170	Same	81
People	624	Son	304	First	161	Few	79
Friend	576	Nurse	303	Submitted	142	Avid	79
March	549	July	298	Amazing	136	Human	77
Child	528	California	290	Old	133	Most	77
Wife	481	Heart	290	Beautiful	119	Grateful	76
@alexgoldstein	452	Mom	278	High	115	Able	74
Time	444	Nov.	274	Young	114	Social	74
Father	441	Virus	268	Big	112	Happy	68
Jan.	439	Death	257	Special	110	Kind	65
School	435	City	254	Hard	108	Strong	63
Texas	396	World	252	Own	107	Sweet	63
Daughter	376	Loss	234	Proud	106	Next	60
Veteran	375	Person	232	Devoted	105	Caring	57
York	374	Hospital	227	Real	105	Favorite	56
Mother	353	Everyone	224	Important	101	Healthy	54

### Nouns

**FIGURE 3:**
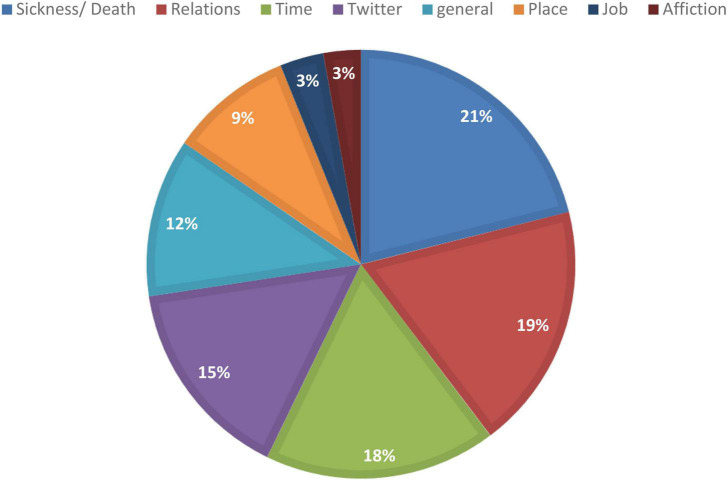
Major semantic categories in the FoC.

The identified nouns could be further grouped semantically into eight major categories, as demonstrated in Figure 3. The chart was created by compiling all of the nouns denoting a particular semantic group in relation to the total number of tokens for the top 50 nouns identified in Table 1 (30,106 tokens). Unsurprisingly, given the focus and scope of the FoC account, nouns related to the pandemic, such as *COVID-19* or *death*, took the lead, accounting for more than 21% of the top 50 nouns. In the second place, at roughly 19%, came words denoting the relations expressed by the tweets to describe the deceased’s relationship with the person submitting the tweet, such as familial terms, *friend*, and *child*. Within this category, the vast majority of words (>90% of cases) signified familial relations, while the remainder referred to a friend. Identification of time and space with relation to the time/place of death, origin, or residence of the deceased was among the major semantic themes, occupying the third (18%) and sixth (9%) places, respectively. In fourth place (15%), there were three nouns bearing strong indications of the microblogging genre on Twitter, i.e., *rt* (meaning a retweet of a previous tweet) and mentions of two Twitter users (@FacesofCovid and @alexgoldstein [the creator of the FoC Twitter account]). A general category was created for words with rather general semantic references, such as *life* and *people*; this category came in fifth place at 12%. The last two categories, job and affection, were included as well, with roughly 3% of related words for each. In the affection category, nouns such as *love* were included, while the job category included nouns such as *veteran, teacher*, or *nurse*.

### Adjectives

The top 50 adjectives were grouped based on their semantic meaning similar to the process conducted in the previous section. The initial analysis of these adjectives and utilization of the *KWIC* tool of #LancsBox revealed that it was possible to identify the adjectives that were explicitly indicative of quantities, e.g., *more, many*, and *few*. These came in second place, with 23% of the 6,945 adjective tokens identified in Table 2. Similarly, it was possible to identify the adjectives related to describing details concerning age and time, such as *old, young*, and *past*, which came in fourth place at 7%. Adjectives with more than one semantic meaning were classified according to their evaluative content. To illustrate, certain words, including *beloved, loving*, and *kind*, were directly assigned positive polarity. However, for many of the adjectives identified on this list, the textual context as expressed in the concordance lines revealed conflicting polarities.

**TABLE 2:** Top content word collocates of she was and he was in the FoC.

He was		She was	
Type	Token	Type	Token
Beloved	192	Beloved	80
Father	116	Nurse	77
Veteran	101	Mother	66
Man	62	Teacher	51
Husband	57	Loving	40
Loving	51	School	36
Retired	51		
Kind	50		
Teacher	47		
Loved	43		
Firefighter	38		
Best	37		
Years	35		
Devoted	33		
Owner	31		

For instance, *big* (112 tokens) as a lemma for big, bigger, and biggest revealed 89 cases with positive polarity signifying big personalities, big hearts, or big presence while the remaining 23 cases were rather relatively neutral as in “big brother.” Similarly, *special* (110 tokens) was used neutrally (57 tokens) and in collocations with special needs, special education, and Special Olympics but was also used positively in the remaining 53 cases. *Social* (74 tokens) revealed similar patterns where it was used neutrally in conjunction with the social worker, social distancing, or social media but also signified positive polarity when acknowledging the lost ones’ social outgoing nature. *Hard* (108 tokens) expectedly signified negative polarity (61 tokens) but also denoted a positive one (47 tokens) when signifying the quality of being hard working. By the same token, *positive* (94 tokens), denoted naturally positive polarity as in positive personality (37 tokens), but given the focus of the FoC, the remaining cases (57 tokens) all referred to a positive COVID-19 test, thus entailing a negative polarity. This positive/negative polarity is also exhibited in *able* (74 tokens) when narrating the morning families’ sorrows for the inability to be by the lost ones in their last moments due to pandemic restrictions or their gratitude for being able to say goodbye. Finally, the *same* (81 tokens) was used primarily neutrally (55 tokens) on the same day but could also signify negative polarity when tweets narrate that things are no longer the same for the mourning families (26 tokens).

**FIGURE 4:**
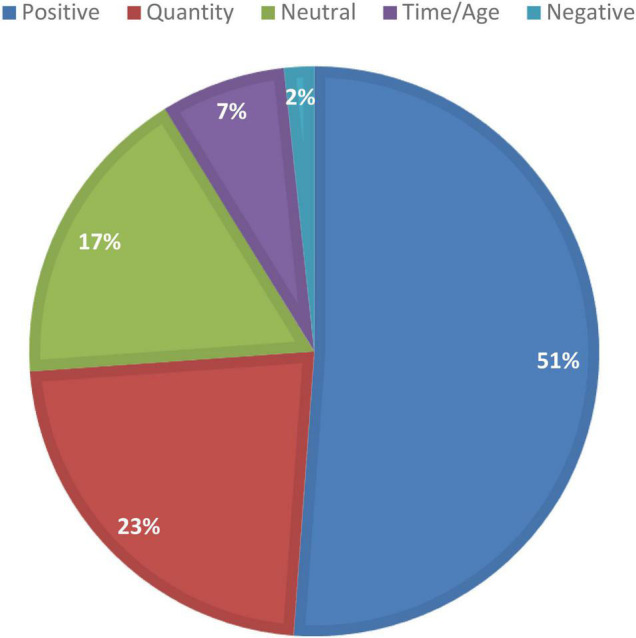
Major semantic categories for the top 50 adjectives in the FoC.

All this was regrouped accordingly into five major areas as in Figure 4 below, which clearly signifies the dominance of positive polarity in more than half of the identified adjectives. However, note that while this analysis attempts to reveal the patterns identified in FoC, corpus analysis in such a way approaches the dataset from a top-down perspective and is better aided by more bottom-up processing to confirm the findings within a more contextualized environment. The following sections offer a micro-level analysis with a special focus on gender differences, if any, in the FoC.

It was evident based on these findings that certain linguistic patterns persisted throughout the entire corpus and were detected in both the examined nouns and adjectives. However, note that in addition to the positive polarity and semantic diversity established in the representation of the deceased throughout the analysis, the findings at this stage clearly signified the interpersonal aspect of the FoC tweets. This was evident in the extensive utilization of many inherent features of microblogging, as discussed in the literature review. Consulting the *KWIC* tool for mentions (@), for instance, resulted in 5,499 tokens, with *@FacesofCovid* (1,645) and *@alexgoldstein* (452) at the top of this list (Table 1). Another repeatedly used inherent feature was the hashtag, whose affordances were discussed earlier in Section 1. More than 460 hashtags were used repeatedly in the FoC tweets, such as *#covid, #covid19*, and *#facesofcovid*. While the intensive use of mentions served to create links within the community established by the FoC account, the use of hashtags had the potential to expand those links outside of that space.

### Stage 2

**FIGURE 5:**
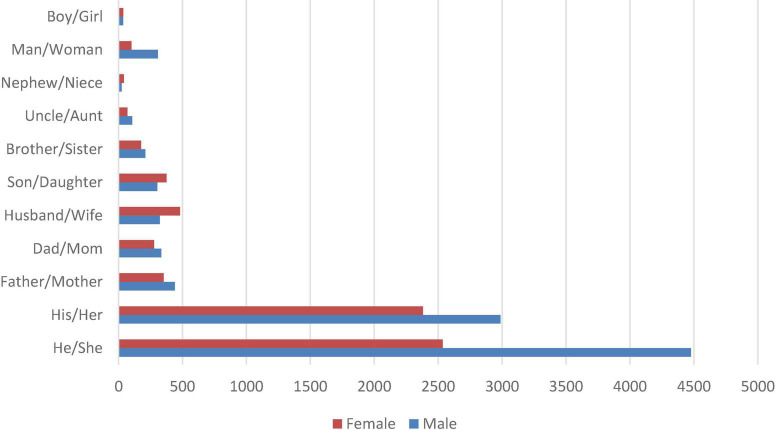
Frequency of gendered terms in the FoC.

With a more specific focus, this stage attempted to identify whether there were any differences in the reporting of the tweets based on the gender of the deceased. However, note that while working in stage 1, function words, such as pronouns, were excluded to allow for a content-based coverage of the patterns expressed in the tweets. This section starts by identifying gendered lemmas and their frequencies. Examination of the frequency list generated by the *Words* tool in Table 2 revealed the profound presence of the third-person masculine and feminine pronouns *he* and *she*, respectively, along with their corresponding possessive pronouns, *his* and *her*, respectively. Figure 5 illustrates the frequency of these lemmas as well as of other gendered words, such as *mother, father, brother*, and *sister*, and how they were distributed. With the exception of *he/she* and *man/woman*, none of the pairs revealed statistically significant differences between the male and female counterparts. However, this should be understood in light of the fact that these terms did not necessarily refer to the deceased; for instance, they could be referring to other relevant family members involved, as in examples 1 and 2 below.

1. She is survived by her parents, her *son*, and her sibling.
2. …., a humorist who was brilliant at political cartoons and was as crazy a dog lover as his *wife*, …

It appeared from this data analysis and corpus processing that stories related to male death cases were submitted more frequently than stories related to female death cases. With this in mind, it was essential to consult the *KWIC* tool to reveal which concordance lines actually described the dead. In doing so, it was evident that the use of a combination of the third-person pronoun and past form of *be* (i.e., *he* + *was* and *she* + *was*) was efficient in generating concordance lines describing the deceased in particular. A *he* + *was* search resulted in 1,839 occurrences, while a *she* + *was* search resulted in 1,003 occurrences. This was consistent with the results found earlier with regard to the number of posted tweets for the two genders. These occurrences were processed by the *GraphColl* tool to generate which collocations occurred most frequently with each structure.

**FIGURE 6:**
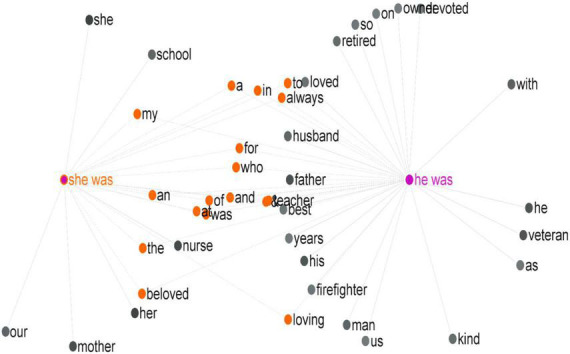
Collocates of she was and he was in the FoC as generated by #LancsBox.

To start, note that not all tweets contained the phrases *he was* or *she was*; however, this construction should provide a representative sample of the tweets. In light of this, the right span in the *GraphColl* tool was set to zero words, while the left was set to five words. Larger numbers resulted in busier graphs that could not be analyzed efficiently. The threshold was set to 30, meaning that a word had to occur at least 30 times in the tweets to be considered a collocate of *he was* or *she was*. Figure 6 demonstrates the 36 collocates of *he was* and the 23 collocates of *she was* as well as their overlapping collocates. The difference in the number of collocates was consistent with the difference in the number of occurrences of *he* + *was* and *she* + *was.*

Focusing on content words only (Table 2), these figures revealed some similarities and differences in the results. For instance, *beloved* dominated these two lists identifying an evaluative adjective along with other affect- and judgment-based modifiers concerning the deceased. Among these, *loving* was dominant within the tweets for both genders, while kind and devoted were identified as collocates with *he* + *was* only. Family roles, e.g., *mother, father*, and *husband*, were also common, as shown in Table 2, which was consistent with the findings of stage 1. Words denoting job descriptions were more prominent in this list compared to those identified in stage 1, which barely comprised 3%. This prominence, in particular, was more evident with *he* + *was* collocates. However, Table 2 confirms a traditional perception of how professions are assigned to the two genders, with *she* + *was* collocating with *nurse* and *teacher* and *he* + *was* collocating with *veteran, firefighter*, and *owner*. The next section targets such a difference from a more specific perspective, adopting social actor representation as a framework for analysis rather than utilizing corpus tools.

### Stage 3

The last stage of the analysis was conducted with a social actor representation perspective, examining 200 random tweets from the FoC account (100 eulogizing men and 100 eulogizing women). To start, note that almost all of the mourning tweets, regardless of the gender of the deceased, followed the same pattern. Examples 3 and 4 below demonstrate a case for each gender, with the proper names identified in the tweets omitted. The tweets started with the full name (given and surname), age, and city/state of origin/residence, followed by the date of death. The remainder of the tweets described the deceased in the language of each tweet’s original writer, i.e., the person sending the content to the account with the intention of sharing it *via* that platform.

1. ^** **^, 70, of ^******^, New Mexico died of COVID on Jan. 29, 2021. He spent 45 years as a heavy equipment operator and loved smoking meat.
2. “Of all the things that ^**^ was, the thing he did the very best was being a father”
3. ^** **^, 79, of Ohio, died of COVID on April 26, 2021.
4. “^**^ loved pretty things and enjoyed shopping for a bargain to decorate her home and tabletops. She also liked watching the Cleveland Browns, Cavs, and Indians and Ohio State Buckeyes”

**FIGURE 7:**
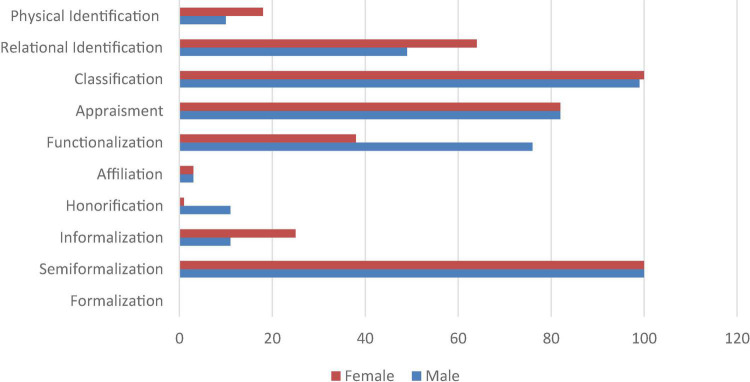
Social actor representation in a random 200-tweet sample from the FoC.

Due to this pattern, it is unsurprising that in almost all of the examined tweets, semiformalization and classification by age, residence, or origin were always present, irrespective of gender. However, informalization with given names/nicknames seemed to be more common for women than for men. On the contrary, honorification, primarily with titles such as *Dr.*, were almost exclusively used for men only. This was primarily linked to the fact that while the use of words denoting jobs was confirmed for both genders in previous stages, the detailed analysis in this section revealed double the functionalization cases in male micro-eulogies (76%) compared to female ones (38%) in this random sample. This differed from the results for relational identification, which appeared to be more prominent in female micro-eulogies (64%) while it occurred in slightly less than half of the male micro-eulogies. Interestingly, physical identification revealed similar patterns favoring female micro-eulogies at 18% of their tweets compared with 10% for the male ones. In all 200 examined tweets, physical identification was used primarily to denote the deceased’s *beautiful smile* or, in fewer cases, to comment on their health conditions prior to their loss. Both appraisement and affiliation were used equally for men and women despite the prominence of appraisement (82% each) compared to the scarcity of affiliation (3% each). Figure 7 summarizes these findings.

This, however, is not to say that similarities were impossible to detect in this analysis. Figure 7 clearly demonstrates areas wherein both men and women had extensively similar representation. This was particularly evident in the case of semi-formalization, whereby all deceased were referred to by their given names and surnames, irrespective of gender, as well as in the case of categorization. Nevertheless, such similarities should be examined in light of the aforementioned fact that all of these mourning tweets conformed to the same pattern of representation. Appraisement, however, which was used at a similar frequency for both genders, represented a solid construction that is usually associated with eulogies. This focus on appraisement provided consistent findings that were established in stages 1 and 2 as well. This suggested that while these stages were separated for the sake of clarity of presentation and analysis, they were approached from an inclusive perspective in search of recurrent patterns. Thus, the findings are discussed collectively in the following section.

## Discussion

Based on the detailed examination of the FoC and considering the RQs, the findings of the three stages of the analysis revealed some consistencies across the linguistic patterns targeted at each stage. To illustrate, the recurrence of the theme of relationships/kinship was established in both the corpus and social actor analyses. This is prominent in the themes identified with the most recurring nouns in the FoC as well as the collocates of *he was* and *she was*. This was unsurprising considering the communicative functions upon which these micro-eulogies were created. Coupled with the highlighted positive polarity associated with the adjective analysis in stage 1, these results were consistent with what has been reported in the previous literature. Hayes (2016) provided evidence from three experimental studies that prove a pervasive motivational tendency in eulogies to positively praise the deceased—and perhaps even idealize them—irrespective of their close or distant relationship. Further, he added that this is the case as it could mitigate some of the distress caused by death-related reasons. Considering RQ1, for instance, it is possible to view FoC, in general, in light of this common theme. This was also evident in the prominence of appraisement for both genders, which could be linked to the gender similarities hypothesis (Hyde, 2005). While the current paper targeted asymmetries in discursive representations of both genders, common areas of construction should not be downplayed or overlooked.

Considering the motivations shared earlier on the creation of the FoC Twitter account, such findings could be linked to system theory (Bronfenbrenner, 1979). Within such a conception, it is possible to envision a system where people’s communicative behavior bonds them; in this sense, parts within that system exist in relation to other parts. This serves to highlight that meaning resides in interactions rather than in people and that it is interactionally constructed (Berger and Luckmann, 1967), which is consistent with the constructionist theories of discourse noted in the introduction. What is created through these tweets—and through the FoC account as a whole—is a virtual system encompassing a virtual community (Al Maghlouth, 2018), where the bereaved look for support and relief and demonstrate what Verma and Neimeyer (2020) classified as collective vulnerability and socially shared grief. In this sense, social media platforms, such as the FoC account, offer a good coping mechanism (Kakar and Oberoi, 2016) in response. The creation of this community has also been supported by the identified inherent features of microblogging, which can be directly linked to the linguistic feature of intertextuality. In intertextuality, texts are linked to other texts or references outside of their immediate linguistic context (Reisigl and Wodak, 2009). With this in mind, such intertextuality serves to intensify the affiliation created within virtual communities (Zappavigna, 2012, 2014), even in its most basic ambient forms.

Regarding the gender-based examination sought in RQ2 and RQ3, interestingly, this particular dataset did conform to the unanimous findings of gender asymmetry presented in the literature on gendered discourse (Pearce, 2008; Baker, 2010, 2014; Baitinger, 2015). In the FoC, men were more present than women, were constructed *via* more functionalization, and had more honorification than women, who took the lead in relational and physical identification instead. Even in eulogies, women were still found to be constructed through rather stereotypical gender representations, which was consistent with the findings of many corpus-based works (Vu et al., 2018). This is interesting because unlike many ideologically—and, perhaps, intentionally—loaded discourses that often target media discourses to examine gender representations, the representations constructed in the tweets were actually produced as micro-eulogies to memorialize the dead by their loved ones. This could suggest that even with the purest intentions, language—natural and authentic language, in particular—appears to be inherently gendered. This finding conforms to the constructionist view of discourse and gender within wider social and cognitive discursive circles. Gender, which has been established repeatedly in the relevant literature works as socially and culturally constructed (Butler, 1999, 2006; Wood, 2004), remains subconsciously confined to linguistic asymmetry. Considering the more inclusive take on gender established in the literature review and expressed through social role theory (Eagly and Wood, 2012), expecting a parallel asymmetry in corresponding discourse does not come across as surprising.

Moreover, due to the constructionist perspective taken and given the affordances of microblogging communication highlighted earlier, it could be assumed that such gendered representations were demolished or at least constrained. To illustrate, social representations—including those pertaining to gender—are inherently dynamic and subject to change in response to new contexts (Cohen et al., 2022). Earlier discussions of social media as a platform for online grief have presumed that its affordances should lead to bias-free constructions since individuals can access these platforms without the interfering mediums of traditional media outlets (Lingel, 2013). A decade on from such assumptions, the results of this study suggest that this is not the case. These findings highlight that social media platforms offer discourse analysts a natural laboratory to investigate linguistic and social constructions (Herdagdelen, 2013) and that, even within such laboratories, dominant ideologies are still transported to the public on both conscious and subconscious levels (Popa and Gavriliu, 2015). This highlights the ongoing need to promote awareness of how gender asymmetry is depicted in language. While redressing gender unbalances cannot be achieved overnight, many diachronic studies have documented some improvement toward this goal. This has been evidenced through several discourse analyses within educational fields (e.g., Wharton, 2005; Lee, 2018); it could also be achieved within other domains.

## Conclusion

Resorting to social media platforms to express grief has been normalized in recent years within various social contexts. In light of this, this paper examined a corpus of micro-eulogies posted online as memorials of those lost to COVID-19. The multi-level analysis revealed several linguistic patterns associated with positive polarity with regard to the deceased, irrespective of gender. It also highlighted the existence of gender asymmetry and stereotypical constructions within the corpus, confirming the tendency established in the vast majority of discourse studies. The study also acknowledged the constructionist theories of gender and grief as social constructs and, consequently, highlighted the need to promote more awareness of women’s constructions in natural language. Based on such theories, the findings implied that this could be an indicator of the inherently gendered nature of language, which continues to be transformed discursively and subconsciously through social interaction. In light of this, further research into discursive studies within eulogy discourse is recommended, especially considering the scarcity of gender-based investigation in this field. Such research should be carried out with an interdisciplinary motivation comprising linguistic, social, and psychological avenues.

This study also illustrated the feasibility of combining corpus tools with other analytical frameworks, such as social actor representation. Despite the potential of corpus processing in providing analysts with an extensive perception of larger sets of natural language data, on its own, corpus linguistics might overlook certain patterns. As a result, the utilization of more bottom-up processing through manual analysis could potentially provide analyses with more depth and intensity. Thus, it could be argued that a multi-level take on methodology should always be encouraged in future research in response to such implications, which is more consistent with the inter- and multidisciplinary nature of discourse studies.

## Data availability statement

The raw data supporting the conclusions of this article will be made available by the authors, without undue reservation.

## Author contributions

The author confirms being the sole contributor of this work and has approved it for publication.
